# Perfusion-Diffusion Ratio: A New IVIM Approach in Differentiating Solid Benign and Malignant Primary Lesions of the Liver

**DOI:** 10.1155/2022/2957759

**Published:** 2022-01-15

**Authors:** Joanna Podgórska, Katarzyna Pasicz, Witold Skrzyński, Bogumił Gołębiewski, Piotr Kuś, Jakub Jasieniak, Anna Kiliszczyk, Agnieszka Rogowska, Thomas Benkert, Jakub Pałucki, Iwona Grabska, Ewa Fabiszewska, Beata Jagielska, Paweł Kukołowicz, Andrzej Cieszanowski

**Affiliations:** ^1^Department of Radiology I, The Maria Sklodowska-Curie National Research Institute of Oncology, Warsaw, Poland; ^2^Medical Physics Department, The Maria Sklodowska-Curie National Research Institute of Oncology, Warsaw, Poland; ^3^Department of Oncology and Internal Medicine, Maria Sklodowska-Curie National Research Institute of Oncology, Warsaw, Poland; ^4^Department of Gastroenterology, Hepatology and Clinical Oncology, Centre of Postgraduate Medical Education, Warsaw, Poland; ^5^Department of Oncological Gastroenterology, The Maria Sklodowska-Curie National Research Institute of Oncology, Warsaw, Poland; ^6^Application Development, Siemens Healthcare GmbH, Erlangen, Germany; ^7^Second Department of Radiology, Medical University of Warsaw, Warsaw, Poland

## Abstract

**Introduction:**

In order to improve the efficacy of intravoxel incoherent motion (IVIM) parameters in characterising specific tissues, a new concept is introduced: the perfusion–diffusion ratio (PDR), which expresses the relationship between the signal *S*(*b*) decline rate as a result of IVIM and the rate of signal *S*(*b*) decline due to diffusion. The aim of this study was to investigate this novel approach in the differentiation of solid primary liver lesions. *Material and Methods*. Eighty-three patients referred for liver MRI between August 2017 and January 2020 with a suspected liver tumour were prospectively examined with the standard liver MRI protocol extended by DWI-IVIM sequence. Patients with no liver lesions, haemangiomas, or metastases were excluded. The final study population consisted of 34 patients with primary solid liver masses, 9 with FNH, 4 with regenerative nodules, 10 with HCC, and 11 with CCC. The PDR coefficient was introduced, defined as the ratio of the rate of signal *S*(*b*) decrease due to the IVIM effect to the rate of signal *S*(*b*) decrease due to the diffusion process, for *b* = 0.

**Results:**

No significant differences were found between benign and malignant lesions in the case of IVIM parameters (*f*, *D*, or *D*^∗^) and ADC. Significant differences were observed only for PDR, with lower values for malignant lesions (*p* = 0.03). The ROC analysis yielded an AUC value for PDR equal to 0.74, with a cut-off value of 5.06, sensitivity of 81%, specificity of 77%, and accuracy of 79%.

**Conclusion:**

PDR proved to be more effective than IVIM parameters and ADC in the differentiation of solid benign and malignant primary liver lesions.

## 1. Introduction

Intravoxel incoherent motion (IVIM) imaging allows the extraction of perfusion data from diffusion-weighted imaging (DWI). It is achieved through an MRI acquisition with multiple small *b*-values and the description of results with a bi- or triexponential function [1]. The theoretical concept of IVIM was first presented by Le Bihan et al. and was initially thought to be a very promising method [2].

The measurement of signal intensity for several low-*b* DWI images permits the observation of signal decay. The decay can be separated into two components. The first component is observed for low *b*-values (range 0-200 s/mm^2^). A fast drop in signal in that range is caused by the rapid flow of water particles contained in blood vessels (microcirculation, IVIM). For *b*-values greater than 200 s/mm^2^, a slower signal decrease is observed. Slower movements of water within the tissue are detected (diffusion), with blood flow not significantly affecting the signal.

A biexponential function allows both segments of the curve decay to be analysed. The fast decay part allows the calculation of two IVIM-specific parameters. The first parameter is the perfusion fraction (PF or *f*), reflecting the pseudodiffusion compartment that is related to microcirculation. The second parameter is the pseudodiffusion coefficient (*D*^∗^, *D*_fast_), representing the velocity of the water particles contained in the microcirculation within the voxel and which is dependent on the characteristic length/timescale of the incoherent motion [2]. The slower decay allows extraction of the diffusion coefficient (*D*, *D*_slow_), which reflects “pure” molecular diffusion.

Despite various IVIM techniques of image acquisition and analysis being implemented, the role of IVIM-related parameters (*D*^∗^, *f*) in liver imaging is still not well established. Consequently, the IVIM technique has not gained wider use in the diagnosis and characterisation of liver lesions to date [3–5]. IVIM imaging and the quantification of liver lesions are particularly challenging due to the prominent movement of the diaphragm and heart. Complex hepatic vascularity further limits the role of this technique in characterising liver tissue. The results of the majority of published studies have not confirmed the superiority of IVIM over conventional techniques, when it comes to the characterisation of liver lesions [6–9].

To date, the mathematical concepts of IVIM have focused mainly on analysis of fitting algorithms, with emphasis on the quality of fit and the accuracy of the obtained parameters. Various algorithms have been proposed, i.e., biexponential analysis segmented fitting and full fitting, triexponential analysis, and IVIM biexponential fitting without *b* = 0 [1, 6, 10–12]. However, each of the models focuses on the separate analysis of fast and slow components of the curve decay.

In order to improve the efficacy of IVIM parameters in characterising specific tissues, we have introduced a new parameter. It not only characterises the fast component of DWI but also describes the relationship between two processes [13]. The perfusion–diffusion ratio (PDR) comprises information from both IVIM and diffusion processes.

As the discrimination of nonsolid liver lesions (cysts or haemangiomas) is achieved in a relatively straightforward manner using T2-weighted images, the main diagnostic challenge is the differentiation of benign and malignant solid liver lesions, especially primary tumours. Hence, the aim of this study is to present the novel IVIM approach and investigate its possible advantages over standard IVIM and ADC approaches for the differentiation of solid primary liver lesions.

## 2. Materials and Methods

### 2.1. Study Population

The study protocol was approved by the local ethics committee, and informed consent was obtained from all patients prior to examination.

**Figure 1:**
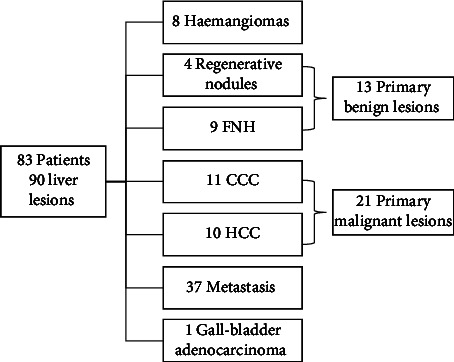
Study population flowchart.

**Figure 2:**
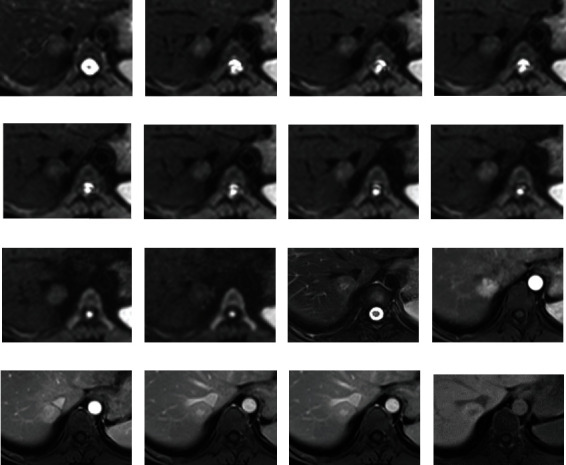
An FNH lesion is shown. Images (a–j) represent subsequent *b*-values in IVIM acquisition; image (k) is T2 haste FS and shows mild hyperintensity of the lesion with a greater signal in the central scar; (l–o) are arterial, portal venous, and equilibrium phase after Gd-Bopta administration and present homogeneous arterial enhancement, with no washout; (p) is the hepatobiliary phase which shows enhancement of the lesion greater compared to the surrounding liver.

**Figure 3:**
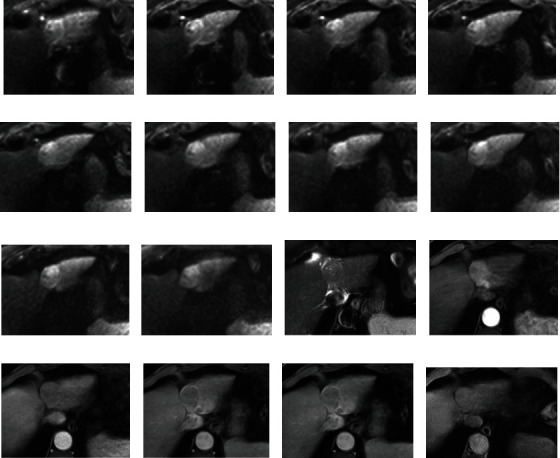
An HCC lesion is shown. Images (a–j) represent subsequent *b*-values in IVIM acquisition; image (k) is T2 haste FS and shows mild hyperintensity of the lesion; (l-o) are arterial, portal venous, and equilibrium phase after Gd-Bopta administration and present arterial enhancement, with washout and capsule appearance; (p) is the hepatobiliary phase which shows enhancement of the lesion similar compared to the surrounding liver.

In this prospective study, 83 consecutive patients referred for liver MRI with a suspected liver tumour and without previous treatment were enrolled between August 2017 and January 2020. Apart from standard liver examination, DWI-IVIM acquisitions using a prototypical sequence were added to the MRI protocol. In total, 90 liver lesions were analysed. Thirty-one lesions were identified as benign, of which 9 were focal nodular hyperplasia (FNH), 4 were regenerative nodules, and 18 were haemangiomas. The diagnosis of these was based on typical appearance on MRI with hepatobiliary contrast, as well as observation over a period of 12-44 months. Fifty-nine of the liver lesions were found to be malignant, with 11 cholangiocarcinomas (CCC), 10 hepatocellular carcinomas (HCC), 37 metastases, and 1 gallbladder adenocarcinoma. In all cases of CCC, the diagnosis was confirmed by biopsy and histopathological examination. In the case of HCCs, the diagnosis was based on LI-RADS criteria [14]: with no histopathological examination in the case of LR5 lesions (*n* = 6) or confirmation by biopsy in the case of <LR5 lesions (*n* = 4). The diagnoses of metastasis were made either based on biopsy (*n* = 34) or by reference to the typical appearance on MRI of colorectal cancer (CRC) metastasis in a patient diagnosed with CRC. In line with these assumptions, haemangiomas and metastases were not included for further consideration by this study. The flowchart describing the study population selection is reported in Figure 1. Examples of IVIM acquisitions are shown in Figures 2 and 3.

Consequently, the final study population consisted of 34 patients with primary solid liver masses. In the case of benign lesions, the patient age range was 44-83 years, with a mean of 54.4 years, while in the case of malignant lesions, the age range was 56-83 years, with a mean of 64.7 years.

### 2.2. The MRI Protocol

MRI examinations were performed on a 3 T MR system (MAGNETOM Skyra, Siemens Healthcare, Erlangen, Germany) with an 18-channel phased-array body coil in combination with the 32-channel spine coil. The detailed MRI protocol is shown in Table 1. For IVIM-DWI, a prototypical free-breathing, single-shot spin-echo echo-planar imaging (SE-EPI) sequence was used with *b*-values of 0, 10, 30, 50, 75, 100, 150, 200, 500, and 900 s/mm^2^. The detailed IVIM-DWI sequence is shown in Table 2. For fat suppression, spectral attenuated inversion recovery was applied. In all cases, hepatobiliary contrast (MultiHance, Bracco Imaging Deutschland GmbH, Germany) was used, with the acquisition of a 70 min delayed phase.

### 2.3. Image Analysis

Image interpretation and data acquisition were accomplished using a scanner dedicated workstation—syngo.via (Siemens Healthcare, Erlangen, Germany). Liver MRI examinations were read by board-certified radiologists (AC, JP, and JP) who specialise in abdominal imaging. For quantitative analysis, three radiologists (JP, 8 y experience in MRI; PK, 3 y experience; and BG, 3 y experience) placed the regions of interest (ROIs) in the liver tumour, avoiding regions of haemorrhage or necrosis. The regions were placed on a b900 image and then copied to the remaining *b*-value images. ROI data entailing mean signal intensity (*S*) values with standard deviations (SD) were then exported for further analysis.

### 2.4. Calculation of DWI and IVIM Parameters

The overall MRI signal attenuation can be described as the sum of the tissue and blood components [2, 15].

(1) $$\begin{array}{c} \frac{S\left(b\right)}{S\left(0\right)}=\left(1-f\right)\cdot e^{\left(-bD\right)}+f\cdot e^{\left(-b\left(D_{b}+D^{*}\right)\right)}, \end{array}$$

where *S* is signal intensity; *b* is a factor depending on the gradient pulse sequence; *f* is the fractional volume of capillary blood flowing in each voxel (ratio of the volume of water flowing in capillary compartment to the total volume of water in the voxel); *D* is the water diffusion coefficient, characterising the mobility of water molecules in the tissue; and *D*_*b*_ is the water diffusion coefficient, characterising the mobility of water molecules in blood; and *D*^∗^ is a pseudodiffusion coefficient, characterising blood microcirculation. *D*_*b*_ is often assumed to be the same as *D* (e.g., in [4]), resulting in.

(2) $$\begin{array}{c} \frac{S\left(b\right)}{S\left(0\right)}=\left(1-f\right)\cdot e^{\left(-bD\right)}+f\cdot e^{\left(-b\left(D+D^{*}\right)\right)}. \end{array}$$

Since *D*^∗^ is higher than *D* or *D*_*b*_ by an order of magnitude, a simplified formula is often used (e.g., in [16]).

(3) $$\begin{array}{c} \frac{S\left(b\right)}{S\left(0\right)}=\left(1-f\right)\cdot e^{\left(-bD\right)}+f\cdot e^{\left(-{bD}^{*}\right)}. \end{array}$$

The simplified formula will be used for the purpose of the study. Two terms on the right-hand side of the equation describe the flow of water in capillary network (fast exponential decay) and outside the capillary network (slow exponential decay). For simplicity, they will subsequently be referred to as perfusion and diffusion, although the first term also includes the diffusion of water in the capillary network (*D*_*b*_ is included in *D*^∗^).

Equation (3) was fitted to signal intensity values to obtain values of *f*, *D*, and *D*^∗^ parameters for each lesion. Gnuplot version 5.0, patchlevel 4, and a nonlinear least-squares (NLLS) Marquardt-Levenberg fitting algorithm were used in the calculations.

The PDR coefficient was introduced and defined as the ratio of the rate of signal *S*(*b*) decrease due to perfusion to the rate of signal *S*(*b*) decrease due to diffusion, for *b* = 0. The rate of signal decrease can be calculated as the first derivative of equation (3), for example.

(4) $$\begin{array}{c} \frac{d\left[S\left(b\right)/S\left(0\right)\right]}{db}=\left(1-f\right)\cdot \left(-D\right)\cdot e^{\left(-bD\right)}+f\cdot \left(-D^{*}\right)\cdot e^{\left(-{bD}^{*}\right)}. \end{array}$$

For *b* = 0, the relationship simplifies to

(5) $$\begin{array}{c} {\frac{d\left[S\left(b\right)/S\left(0\right)\right]}{db}}_{b=0}=\left(1-f\right)\cdot \left(-D\right)+f\cdot D^{*}. \end{array}$$

The first term on the right-hand side of equation (5) refers to diffusion, and the second to perfusion. Thus, PDR can be calculated as:

(6) $$\begin{array}{c} \text{PDR}={\left(\frac{{dS}_{\text{IVIM}}\left(b\right)}{db}/\frac{{dS}_{\text{difussion}}\left(b\right)}{db}\right)}_{b=0}=\frac{f}{1-f}\cdot \frac{D^{*}}{D}. \end{array}$$

Values of *f*, *D*, and *D*^∗^ parameters were used to calculate the PDR coefficient for each lesion according to equation (6).

For the values *b* = 0, 500, and 900 (s/mm^2^), the ADC parameter was calculated from the relationship.

(7) $$\begin{array}{c} \frac{S\left(b\right)}{S\left(0\right)}=e^{\left(-bADC\right)}. \end{array}$$

### 2.5. Statistical Analysis

Statistical calculations were performed using the R environment (version 3.3.2, The R-Foundation, Austria) [17]. Differences at *p* < 0.05 were considered significant. The normality of the data was checked using the Shapiro-Wilk test. IVIM parameters, ADC, and the PDR value obtained for benign lesions were compared with the values for malignant lesions using the Wilcoxon rank sum test. For the PDR coefficient, an ROC curve (AUC) was plotted and the area under the curve, the probability value, accuracy, and cut-off points were determined. The cut-off points were determined using the Youden criterion [18].

## 3. Results

The group of benign liver lesions (*n* = 13) consisted of 9 FNH and 4 regenerative nodules, while the group of malignant lesions (*n* = 21) comprised 11 CCC and 10 HCC lesions.

**Figure 4:**
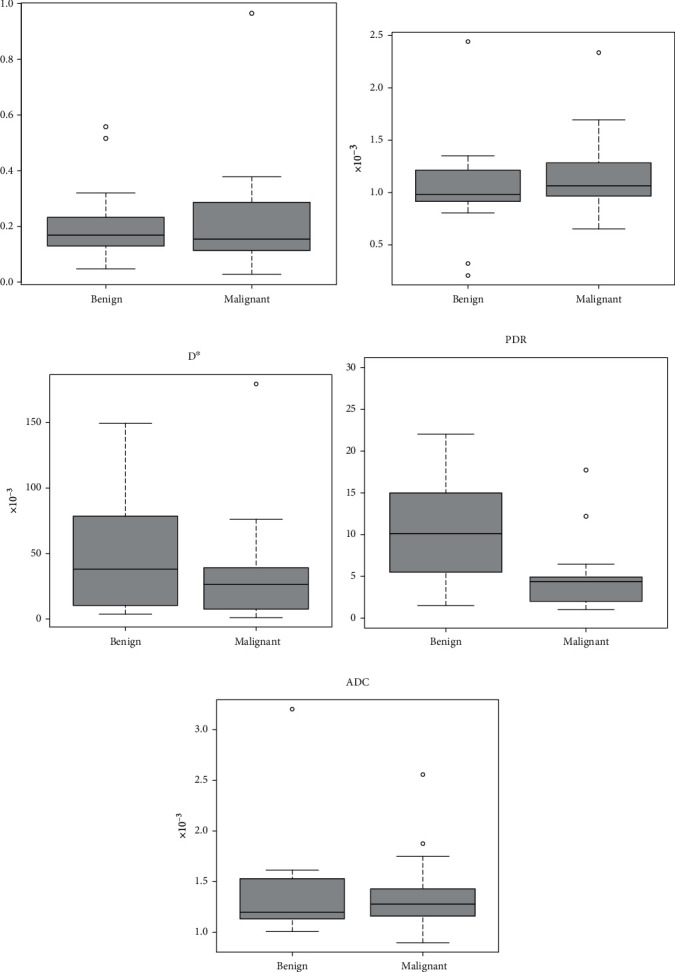
Box-and-whisker plots illustrate the median, interquartile range, minimum, maximum, and outlier data for all measured lesions: (a) *f*, (b) *D*, (c) *D*^∗^, (d) perfusion-diffusion ratio (PDR), and (e) ADC (0, 500, and 900). Significant differences between benign and malignant lesions were only observed for the PDR parameter.

No significant differences were found between the benign and malignant lesions when it came to either the IVIM parameters (*f*, *D*, or *D*^∗^) or ADC. Significant differences were only observed for PDR, with lower values for malignant lesions (*p* = 0.03). The results are presented in Table 3 and Figure 4.

**Figure 5:**
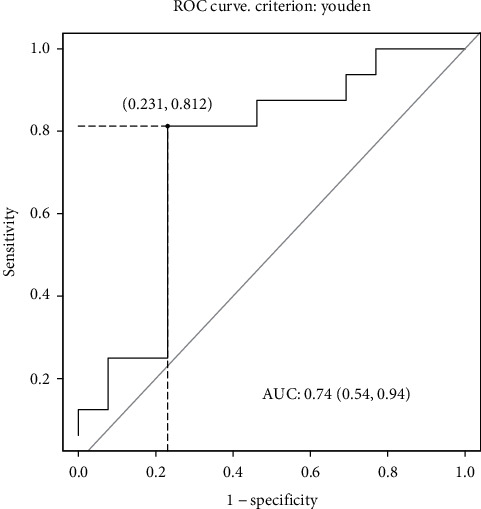
Receiver operating characteristic (ROC) curve for differentiation between benign and malignant liver lesions using the PDR parameter.

The ROC analysis yielded an AUC value for PDR equal to 0.74, with an optimal cut-off value of 5.06, sensitivity of 81%, specificity of 77%, and accuracy of 79% (Figure 5).

## 4. Discussion

In this prospective pilot study, we assessed the efficacy of a new IVIM-related parameter. The PDR characterises the relationship between the IVIM and DWI curve segments, in this way differing from previous concepts that considered the two phenomena separately. In this study, as opposed to classic ADC and known IVIM parameters, PDR allowed for differentiation between solid malignant and benign primary tumours of the liver.

The PDR coefficient was introduced as the ratio of the rate of decline of signal *S*(*b*) as a result of IVIM to the rate of decline of signal *S*(*b*) due to diffusion, for *b* = 0. While *f* is the fractional volume of water flowing in capillary compartment and *D*^∗^ is associated with the velocity of the water particles in capillaries, relative blood flow can be estimated from the product *fD*^∗^ [2]. That said, PDR can be understood as the ratio between the flow of water in the capillary network and outside the capillary network. The advantage of PDR is that it combines all of the IVIM and DWI parameters in one, meaning that if any of the DWI or IVIM parameters are changed, the PDR is also changed. In other words, a statistically significant difference in any of the *f*/*D*/*D*^∗^ parameters may be associated with a statistically significant difference in PDR. At the same time, nonsignificant differences in IVIM and DWI parameters may result in a significant difference in PDR, as observed in the current study.

For the purpose of this study, we used equation (3) to describe the dependence between signal intensity and *b*-values. Other forms of equation were also used, with examples given in equations (1) and (2). Some researchers use other nomenclature for parameters, e.g., *D*_*t*_ and *D*_*p*_ [5] or *D*_fast_ and *D*_slow_ [6]. It should be kept in mind that different forms of the formula are used to describe the diffusion and IVIM phenomena, with an obvious impact on the definition of PDR.

Over the last decade, numerous researchers have sought to cope with the interesting concept of IVIM and found that it is difficult to do in practice. The abdominal region is especially challenging in this respect, given its susceptibility to artifacts. In most studies, IVIM parameters failed to achieve a better diagnostic ability than ADC, giving no justification for the use of the sequence in clinical practice [3].

In a similar study group, Klauss et al. found no significant difference for *f* and *D*^∗^ in differentiating between HCC and FNH, with only *D* and ADC allowing for the differentiation of these two lesion types [4]. Similarly, as Luo et al. sought to distinguish HCC and FNH, they noted statistically significant differences between them in ADC and *D* values. The IVIM parameters, *D*^∗^ and *f*, revealed no significant differences, and *D*^∗^ was further regarded as only poorly reproducible [16].

When it comes to parameters such as IVIM, the technical characteristics of the acquisition are clearly crucial. Several studies have shown that a significant impact on the results obtained is exerted, not only by the fitting algorithms but also by the number of *b*-values used [1, 6, 19]. The greater the number of *b*-values, the better the fit of the function, and the more stable the parameters.

Sokmen et al. found that *f* performed well in differentiating high- and low-grade HCCs, proving to be a slightly more powerful discriminant than ADC (AUC values of 0.866 versus 0.857). What is important here is that their acquisition was achieved with 16 *b*-values, compared to the 10 used by us. However, the difference between the AUC of *f* and ADC was very small [20]. In contrast, in a similar study, Woo et al. failed to obtain statistically significant results for *f* obtained from an 8 *b*-value acquisition. Only the ADC revealed significant differences between HCC grades, in line with the results of Sokmen et al. [3, 20].

Furthermore, a meta-analysis of the use of DWI and IVIM in HCC grading showed the usefulness of *D* and ADC, but not IVIM parameters [21].

The limitations of the study are primarily focused on the small group of patients included. Initially, the prospectively enrolled study group was larger. The focus was narrowed to only primary solid liver lesions, which led to a substantial reduction in the number of cases available for study. Ten *b*-values seemed reasonable, in line with both the data obtained from the literature and our previous experience; however, the acquisition of more *b*-values would probably have improved the function fitting and thus reduced the uncertainty associated with the results. Sequence artifacts, mainly affecting the liver's left lobe, were also problematic. We did not investigate the effect of different equation forms or different fitting algorithms, which could affect the results [6, 10–12]. Additionally, the intersystem reproducibility of the results may be an issue, as diffusion-weighted imaging (DWI) parameters are known to be dependent on the manufacturer/model of the scanner, sequence implementation, and gradient nonlinearity (GNL) [22–24].

In summary, there are numerous existing studies suggesting that *D* is the only IVIM parameter that is useful for the differentiation of liver tumours. Equally, it is worth recalling how *D* reflects the same phenomenon as ADC but does not carry additional information on tissue perfusion. Although *D* is calculated via a biexponential model, as opposed to ADC via a monoexponential model, the relationship between the two is close. Two IVIM perfusion-related parameters (*f* and *D*^∗^), analysed separately, are not useful for the differentiation of liver tumours. For this reason, we propose the use of a PDR parameter, which may prove more effective given the inclusion of both perfusion and diffusion.

The proposed PDR parameter combines all IVIM parameters in one. Our approach, alongside the standardisation of the acquisition and calculation of IVIM parameters, may prove helpful for the introduction of IVIM into clinical practice. It is of course necessary to confirm our results using a larger group of patients. Should it prove useful for liver imaging, the new approach could also be tried in other anatomical regions and clinical situations.

## Figures and Tables

**Table 1:** Acquisition parameters of liver MRI parameters (except for the DWI sequence).

Sequence	Plane	TR	TE	Slice thickness	Breath
Trufi	Coronal	505	1.7	5.0	BH
T2 Haste FS	Coronal	1220	103	5.0	MBH
T2 Haste	Axial	1600	91	5.0	MBH
T2 Haste	Axial	1600	201	5.0	MBH
T2 Blade FS	Axial	6687	90	5.0	RT
T2 Haste FS	Axial	1900	91	5.0	MBH
VIBE Dixon	Axial	3.3	1.3	3.0	BH
TWIST VIBE Dixon + C	Axial	3.9	2.5	3.0	BH
TWIST VIBE Dixon + C	Coronal	4.2	2.6	1.5	BH

FS: fat suppressed; TR: repetition time; TE: echo time; BH: breath hold; MBH: multibreath hold; RT: respiratory triggered; +C: contrast enhanced.

**Table 2:** Detailed acquisition parameters of the IVIM-DWI sequence.

Acquisition parameters	
TR	6100 ms
TE	56 ms
Slice thickness	5 mm
Number of slices	33
Filter	Moderate
Bandwidth	2298 Hz/px
*B* values (s/mm^2^)	0, 10, 30, 50, 75, 100, 150, 200, 500, 900
NSA	3-6
Breath	Free

**Table 3:** ADC and IVIM-derived including PDR in the differentiation of benign and malignant liver lesions (median ± the standard deviation).

Parameter	Benign	Malignant	*p* value
*f*	0.17 ± 0.16	0.15 ± 0.22	0.80
*D*	0.98 ± 0.54	1.06 ± 0.36	0.35
*D* ^∗^	38 ± 48	26 ± 39	0.22
PDR	10.1 ± 7.3	4.4 ± 4.3	0.03
ADC_0-500-900_	1.20 ± 0.58	1.28 ± 0.38	0.90

## Data Availability

The data used to support the findings of this study are available from the corresponding author upon request.
